# Treatment of Pneumonitis

**Published:** 1874-05-01

**Authors:** A. Hermann

**Affiliations:** Pesth


### TREATMENT OF PNEUMONITIS.

BY DR. A. HERMANN, OF PESTH.

*Translated for* THE EXAMINER, *from the Allgemeine Wiener Med. Zeitung, by* H. GRADLE, M.D.

(Concluded from Number VIII.)

THE necessity for caution, on which Juergensen lays especial stress, though he claims to have had no accident as yet, is clearly indicative of the doubts which the originator of the method himself still entertains as to its safety. However, as all potent remedial measures are not without danger, when incautiously employed, this risk ought not to deter us from its employment, were its results satisafctory. Juergensen counted, amongst 200 patients treated according to his notions, 24 deaths, or 12 per cent. mortality; and this statement, if taken unqualified, shows better success than Dr. Hermann's figures of 15.34 per cent. fatality. But amongst this number there were but 32 individuals above 50 years of age, while the statistics of our author show 33 in 163 patients as having passed this *quasi*-critical period; so that the previously detailed influence of age comes here into play in Juergensen's favor. Unfortunately, the analysis is limited to this factor, the seat of the lesion not being specified; and this neglect alone would justify Dr. Hermann in concluding that his cases were of a graver, more dangerous character, whence the augmented mortality.

This conclusion is further confirmed by the short duration of some of Juergensen's cases, as he claims to have seen the disease disappear in twenty-four to thirty-six hours. These deductions, as well as the actual fact that of 127 patients below 50 years of age, Dr. Hermann lost but 7, or 5.5 per cent., while in spite of cold baths and quinine, 168 cases of Juergensen's, of the same description, resulted in 14 deaths, or 8.3 per cent. fatality, suffice to convince the unbiased observer of the inefficacy of this therapeutic method, at the same time that he recognizes its danger *per se*, and impracticability in private practice, where old, deep-rooted preprejudices bar the way to such heroic measures.

Dr. Hermann's own estimation of any treatment of the disease in question he admits, candidly, to be very low. The seat of the lesion determining the gravity of the affection we cannot alter; nor can we control the age or its synonym—individual powers of resistance—as to lose its influence on the tendency to fatal termination. Equally difficult is the limitation of the exudative process, the possibility of which, though often asserted, has never yet been definitely proven, while the reduction of the other most prominent appearance, the pyrexia, has so far remained an unsuccessful attempt, be the *modus operandi* venesection or cold affusion; so that, at present, a purely expectative mode of treatment is the one most justified by past experience, and exclusively adopted by our author, with perhaps the following specification:

As soon as the disease is recognized as pneumonitis, without regard to the lung affected, six ounces of emulsio amygdalina, with one and a half grains of extract. opii aquosum is prescribed, in the dose of one tablespoonful per hour, though discontinued during the patient's sleep. This prescription may be maintained on about the following arguments: The emulsion of almonds quenches the thirst, cooling the dry, hot buccal lining, while the small proportion of prussic acid lessens bronchial irritation, perhaps acting as an anodyne. The opiate, on the other hand, reduces the tendency to cough, quiets the stinging pains, calms dyspnœa, and thus indirectly exerts its soporific influence. Thirst is quenched, besides, by fresh water, whatever the season; while the only nourishment consists in soup, three daily rations of which, however, are but rarely accepted by the patient before improvement has begun; and only after normal temperature and pulse have existed three days, is more solid food, as preserves, permitted, to be changed, after two or three days more, to beef and vegetables, with white bread.

Quite marvellous appears the class of patients under Juergensen's care, who, as he states, were fed during the febrile condition with bread and butter and roast meat, with a liberal daily allowance of wine or beer. That healthy persons should thrive under such treatment is evident; but strange it seems, that so many patients suffering from inflammation of the lungs should possess such healthy appetites, and still prefer hospital residence to their private homes and occupations. Were these light cases an accidental occurrence, or the consequence of hydropathic treatment? But baths will not prevent a fatal issue; nor is their reducing influence on the temperature permanent, as the clinical history of the disease in Juergensen's own daughter sufficiently proves; for, as he says, "The temperature rose above 106° F., and returned to this point so soon after a bath of 60° F. that I concluded to reduce this to 40 to 45° F. and prolong its duration to ten minutes."

When the pulse was observed to become more frequent and smaller, and animal heat diminished, symptoms indicative of paralysis of the nervous centers, recourse was also had to stimulants, especially camphor, *but never with any success, such cases being always fatal.* Thus Dr. Hermann considered it neither excuse nor consolation, when able to say that treatment was commenced *too late;* as such cases fared no better when treated early. When Juergensen, nevertheless, says, concerning the use of stimulants in pneumonitis, that their proper and bold administration, even after incipient insufficiency of the heart, may often prolong life for three or four days, this statement holds good as regards younger individuals, of whom 42 per cent. died during the second week, while but 27 per cent. of the more aged patients survived the first; though this fact is independent of remedial measures, being determined only by the greater powers of resistance during youth; since in our author's cases camphor was administered in the proportion of three to four grains to six ounces of emulsion, while the ten times larger doses of Juergensen accomplished no greater prolongation of life.

Even in the winter season, the patients were exposed to fresh air coming through the windows, opened at least one hour every morning, without any injurious consequences whatever; and as free ventilation is of the utmost importance in a disease of itself interfering with respiration, as pneumonitis, it is certainly safer to endanger its victims by draught than by a vitiated atmosphere.

In conclusion, the author apologizes for an article that can lay no claim to originality; but, as truth is the object of science, and such only has been recorded, he hopes not to have entirely missed his purpose, as he, at least, is well satisfied with the treatment recommended.

